# Development of an Extraction Method of Aflatoxins and Ochratoxin A from Oral, Gastric and Intestinal Phases of Digested Bread by In Vitro Model

**DOI:** 10.3390/toxins14010038

**Published:** 2022-01-04

**Authors:** Paula Llorens, Renata Pietrzak-Fiećko, Juan Carlos Moltó, Jordi Mañes, Cristina Juan

**Affiliations:** 1 Laboratory of Food Chemistry and Toxicology, Faculty of Pharmacy, University of Valencia, 46100 Burjassot, Spain; paullo3@uv.es (P.L.); j.c.molto@uv.es (J.C.M.); jordi.manes@uv.es (J.M.); 2 Department of Commodities and Food Analysis, Faculty of Food Sciences, University of Warmia and Mazury, 10-719 Olsztyn, Poland

**Keywords:** bioaccessibility, validation, aflatoxins, ochratoxin A, bread, *Cucurbita Maxima Pepo*

## Abstract

Validated extraction methods from in vitro digestion phases are necessary to obtain a suitable bioaccessibility study of mycotoxins in bakery products. The bakery industry produces bread with different ingredients to enrich the nutritional properties of this product and protect it from fungal growth. This bread can be contaminated by AFB_1_, AFB_2_, AFG_1_, AFG_2_ and OTA, so an extraction method was developed to analyse these five legislated mycotoxins in digested phases of two types of bread, one with wheat and the other with wheat and also enriched with *Cucurbita Maxima Pepo* at 20%. The studied “in vitro” digestion model consists of oral, gastric and duodenal phases, each one with different salt solutions and enzymes, that can affect the extraction and most probably the stability of the mycotoxins. The proposed method is a liquid–liquid extraction using ethyl acetate by extract concentration. These analytes and components have an important effect on the matrix effect (MEs) in the analytical equipment, therefore, validating the method and obtaining high sensitivity will be suitable. In the proposed method, the highest MEs were observed in the oral phase of digested pumpkin bread (29 to 15.9 %). Regarding the accuracy, the recoveries were above 83% in the digested duodenal wheat bread and above 76 % in the digested duodenal pumpkin wheat bread. The developed method is a rapid, easy and optimal option to apply to oral, gastric and duodenal phases of digested bread contaminated at a level of established maximum levels by European legislation (RC. 1881/2006) for food.

## 1. Introduction

The natural presence of aflatoxins (AFB1, AFB2, AFG1 and AFG2) and ochratoxin A (OTA) in food and raw materials (wheat, oat, rye, barley, grapes) and in their products (bread, biscuits, beer, wine) is still a food safety problem as indicated by the Rapid Alert System for Food and Feed (RASFF) in the last Annual Report 2020. Mycotoxins are in the top 10 of hazards and product categories on food products, aflatoxins and OTA being the most notified mycotoxin hazard [1].

There is a wide variety of food matrices in which mycotoxins have been present, which can have a very significant effect on the mycotoxins’ bioaccessibility, due to the interactions that can occur between the mycotoxin and the food matrix. Although absorption and metabolism depend mostly on the specific properties of the toxin and animal physiology, it is assumed that food matrices maintain a certain influence on these processes. It could interfere with the process of absorption in the intestinal tract. Therefore, the bioaccessibility of a mycotoxin may differ depending on the food considered [2,3]. 

Cereals are very susceptible to contamination of mycotoxins and many studies confirm their presence in various types of cereals (mainly maize, rice, wheat) [4,5]. Cereal products such as bread can be contaminated from the raw material because it is a perishable product, mainly affected by species of the *Penicillium* and *Aspergillus* genera. Both are mycotoxin-producing fungi, including AFB_1_ and OTA [6]. Aflatoxins (B_1_, B_2_, G_1_ and G_2_- mainly) and OTA exert toxic effects in animals and humans. OTA is nephrotoxic, hepatotoxic, teratogenic, neurotoxic, and genotoxic, among other effects [7]. Among all aflatoxins (AFs), AFB_1_ is the one with the highest toxicity and the most potent hepatocarcinogenic, the International Agency for Research on Cancer classifies it as carcinogenic [8]. The production of bread that is most resistant to fungi colonisation with a high nutritional value is the one most desirable goal in the product/cereal industry. In this case, pumpkin is a valuable vegetal rich in carotenoids (vitamin A) and valuable antioxidant compounds, that can protect against the mycotoxins effect and maybe create an antimicrobial product, a most resistant bread. Other authors have studied products such as walnuts (*Juglans regia* L.) with antimicrobial effects [9], zerumbone isolated from ginger rhizomes (*Zingiber officinalis*) with a protective effect against zearalenone [10], or flavokawain A isolated from kava (*Piper methysticum Foster*, *Piperaceae*), which has an anti-apoptotic effect against OTA [11].

Due to the harmful effects of mycotoxins in humans, national and European legislative institutions have established maximum tolerable levels of the sum of AFs, AFB_1_ and OTA in bakery products to protect consumers from the health risks associated with their intake. The highest allowed content for these mycotoxins in food products such as cereals is established in the EC Regulation No. 1881/2006 [12]. 

Consumption of food is considered a major route of exposure to many contaminants, including mycotoxins. Bioaccessibility of mycotoxins is a very important factor determining human health risk assessment [13,14]. 

The effect of a mycotoxin depends on the amount available and its access to the target organ since only a part of the amount ingested will be bioavailable. Bioavailability includes three processes and the bioavailability studies can be performed in vitro using a system that simulates the physiological conditions of the gastrointestinal tract. These systems are a good alternative to in vivo models due to their low cost and lower variability. Although, it is not possible to fully reproduce the physiological conditions and the application of the results is more limited than in the in vivo methods. In recent years, the use of cell cultures was widely used to assess bioavailability and the Caco-2 cell monolayer is widely accepted by pharmaceutical companies and authorities as a standard test for predicting the intestinal permeability of a compound [15].

Quantification of bioaccessibility of a compound, such as mycotoxins, from a certain matrix, is difficult and often hampered by complex processes comprising digestion. Furthermore, the matrix difference has a considerable influence on the recoveries obtained and consequently on the reliable results [16]. Liquid chromatography coupled to MS is usually used for the determination of mycotoxins in bioaccessibility and bioavailability studies following the extraction, using the same procedures that were used for the analysis of mycotoxins in food [17,18,19,20]. Therefore, an efficient and studied procedure extraction is required. Few of the proposed methods in the literature include a complete validation method in the three phases of digestion and it is of great importance to perform it, due to it potentially resulting in a reliable quantification [16].

The present study aims to investigate a liquid–liquid extraction method to evaluate the bioaccessibility of AFs and OTA from two different types of bread, one being a common bread with wheat and the other which contained a percentage of pumpkin, using an in vitro digestion model under fed conditions and evaluating the efficiency of extraction in oral, gastric and duodenal phases of digestion.

## 2. Results and Discussion

### 2.1. Fortification Levels of OTA and AFs Analysis

In all products derived from unprocessed cereals, including processed cereal products and cereals intended for direct human consumption, European legislation has established the maximum levels (ML) for OTA, AFB_1_ and the sum of AFs at 3 μg/kg, 2 μg/kg and 4 μg/kg, respectively; for unprocessed cereals at 5 μg/kg for OTA; however, in processed cereal-based foods and baby foods for infants and young children for OTA and AFB_1_ it is 0.5 and 0.1 μg/kg, respectively [12]. Despite these regulations and food controls, in routine customs analysis and on products from various countries, levels of these mycotoxins were detected in bread. Recently, in the literature, positive bread samples from Guyana [21], Morocco [22], Portugal [23,24], Turkey [25], and Malaysia [26] were registered. These levels ranged from 0.03 to 149 μg/kg for OTA and from 0.05 to 4.9 μg/kg for AFB_1_. Surprisingly, it is possible that some consumers can intake these mycotoxins through one of the most consumed products such as bread and, in some cases, 30 times above the ML. 

These levels are ingested and they could be reduced after the digestion process, as other authors have indicated with food and feed [3], furthermore, the bioaccessibility of mycotoxins depends on the toxin considered and the food matrix in which experiments are carried out. In fact, the bioaccessibility of OTA has proven to be very variable; values near 100% were shown [27], but also below 30% [28]. On the other hand, it is interesting to consider the analytical aspects during the extraction and detection, which can reduce the values down by 10–20% of the real value, due to the matrix effect and sensibility of the equipment.

According to that, the studied levels of fortifications were at three levels (2.5, 5 and 10 μg/kg, level L1, level L2 and level L3, respectively). Fortifications were made after the preparation of bread, after the digestion process adding 0.25 mL, 0.5 mL, and 1 mL of the working solutions (100 µg/mL) to 10 g of wheat bread. 

### 2.2. Validation Results Analysis

#### 2.2.1. Linearity and Sensitivity (LOD and LOQ)

Linearity was evaluated with calibration curves, which were constructed for each mycotoxin and a blank sample at concentration levels ranging from 0.01 µg/mL to 1 g/mL. Different calibration curves were constructed for each phase of digestion (Table 1). All studied mycotoxins presented good linearity, with R^2^ ranging between 0.9986 to 0.9999.

To evaluate the sensitivity of the method, LODs and LOQs were determined by the signal-to-noise ratio (S/N) ≥ 3 and ≥ 10, respectively, from chromatograms of samples of digested wheat bread spiked at the lowest level validated, and using the chromatogram of quantitating ion product. LOQs displayed in Table 2, ranged from 2.01 to 3.51 ng/g. AFB_1_ had the lowest LOQs, which was important since this is a potent liver carcinogen. LOQs were compared with MLs for products derived from cereals set out in Commission Regulation (EC) No. 1881/2006 [12]. None of the mycotoxins exceeded the MLs defined for regulated mycotoxins, the LOQ values ranged from 1.03 to 2.34 ng/g (Table 1). This is the first study that evaluates this parameter in the three digestion phases with bread. Raiola et al. [29] evaluated AFB_1_ and OTA in pasta only in the duodenal phase and observed LOQ values lower than ours 0.15 ng/g for OTA and 0.3 ng/g for AFB_1_; however, Saladino et al. [14] observed similar values of LOQ of AFB_1_ (0.75 ng/g) in duodenal digested bread and a higher result for AFB_2_ (3.35 ng/g) than ours. 

**Table 1:** Linearity and sensitivity data of the extraction method in three digested phases of wheat bread and pumpkin bread.

Bread	Mycotoxin	Oral				Gastric				Duodenal			
		LOD (ng/g)	LOQ (ng/g)	Calibration	R^2^	LOD (ng/g)	LOQ (ng/g)	Calibration	R^2^	LOD (ng/g)	LOQ (ng/g)	Calibration	R^2^
WHEAT BREAD	AFB_1_	0.50	1.66	y = 0.0006x − 2.3155	0.9998	0.51	1.70	y = 0.0156x − 1.4406	0.9999	0.48	1.60	y = 0.0006x − 1.0222	0.9999
	AFB_2_	0.36	1.19	y = 0.0098x + 0.371	0.9989	0.61	2.04	y = 0.0024x − 31.199	0.9961	0.34	1.14	y = 0.0095x + 0.407	0.9989
	AFG_1_	0.43	1.43	y = 0.001x − 0.3127	0.9992	0.60	2.01	y = 0.0088x + 0.398	0.9989	0.41	1.38	y = 0.001x + 0.0547	0.9994
	AFG_2_	0.63	2.09	y = 0.0045x − 0.4783	0.9997	0.91	3.04	y = 0.0021x − 0.4147	0.9992	0.60	2.02	y = 0.0043x − 0.3119	0.9997
	OTA	0.37	1.24	y = 0.0012x + 0.0101	0.9999	0.73	2.44	y = 0.0045x − 0.5683	0.9997	0.36	1.20	y = 0.0011x − 0.0378	0.9988
PUMPKIN BREAD	AFB_1_	0.52	1.74	y = 1595.9x + 3720.1	0.9995	0.54	1.79	y = 422.1x + 14088	0.9996	0.47	1.58	y = 1666.7x + 1701.4	0.9996
	AFB_2_	0.37	1.25	y = 99.215x − 32.625	0.9986	0.64	2.14	y = 984.96x + 2979.2	0.9978	0.34	1.13	y = 105.26x − 42.784	0.9986
	AFG_1_	0.45	1.50	y = 978.79x + 336.58	0.9989	0.63	2.11	y = 1275.1x + 63547	0.9960	0.41	1.36	y = 1000x − 54.626	0.9991
	AFG_2_	0.66	2.19	y = 219.2x + 107.6	0.9994	0.96	3.19	y = 784.6x + 43156	0.9989	0.60	2.00	y = 232.56x + 72.437	0.9994
	OTA	0.39	1.30	y = 818.18x − 8.2376	0.9996	0.77	2.57	y = 64.262x + 94.415	0.9994	0.36	1.19	y = 870.03x + 37.897	0.9985

LOD: limit of detection; LOQ: limit of quantification; R^2^: coefficient of determination.

#### 2.2.2. Precision and Trueness

**Figure 1:**
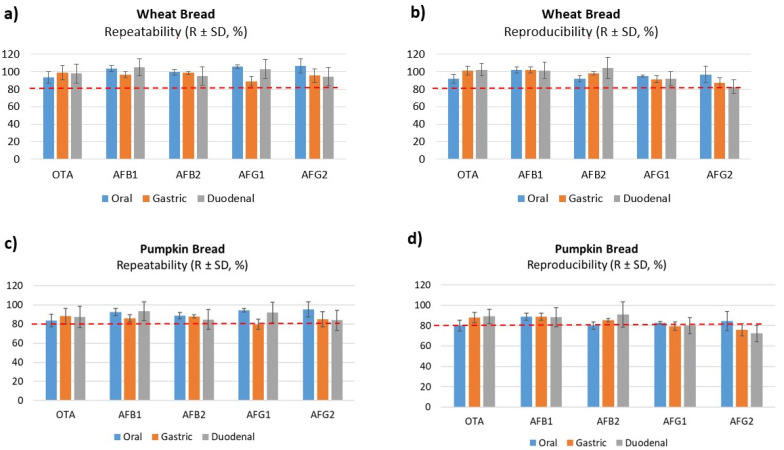
Accuracy (repeatability and reproducibility) data obtained with digested of wheat bread (**a**,**b**) and pumpkin bread (**c**,**d**) at 5 µg/kg (level L2).

The precision of the method was evaluated based on relative standard deviation (RSDs) of repeatability and within-laboratory reproducibility. Considering all concentration levels, maximum values were 18% for repeatability and 12% for within-laboratory reproducibility, for values observed in digested duodenal wheat bread, as shown in Table 2. Results showed no concentration-dependent differences and fulfilled performance criteria for regulated mycotoxins. In Figure 1, the precision and trueness in the three phases studied is shown, and no significant differences between oral, gastric and duodenal phases were observed, however, a decrease in the recoveries was presented that will be due to the increased food breakdown and digestion and the enzymatic activity. Other authors have indicated RSDs values in different duodenal matrices and all of them were close to those observed in our study. Raiola et al. [29] obtained 4.24% and 9.19% for AFB_1_ and OTA, respectively, in pasta; Saladino et al. [14] validated AFB_1_ and AFB_2_ in the duodenal digested phase of bread and observed RSDs of 3.4% and 4.2%, respectively.

**Figure 2:**
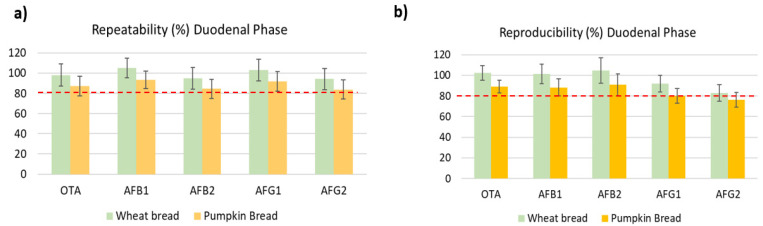
Comparing repeatability (**a**) and reproducibility (**b**) of used method in digested wheat bread and pumpkin bread samples.

Trueness was determined based on the average and RSD of recoveries. Recoveries were assessed using a fortified digested blank matrix. Overall, recoveries varied from 83 to 110% for digested duodenal wheat bread and were within guideline ranges set in the Decision Commission (EC) No. 657/2002 for all analytes (Table 3) [30]. The values for digested pumpkin bread were lower than wheat bread (Figure 2), this decrease was similar in all three phases. Massarolo et al. [20] studied the AFs bioaccessibility from wheat bread but no results of the validation method were carried out in the three phases or included in the digested phases. Other studies that analyzed other mycotoxins in crispy bread and wheat bread observed lower values than ours. De Angelis et al. [18] obtained a recovery of 60–86% in gastric and 70–90% intestinal with wheat bread of T-2 and HT-2, however, Meca et al. [31] analyzed ENs only in the duodenal phase in crispy bread obtaining values below 89%. 

#### 2.2.3. Matrix Effect

**Figure 3:**
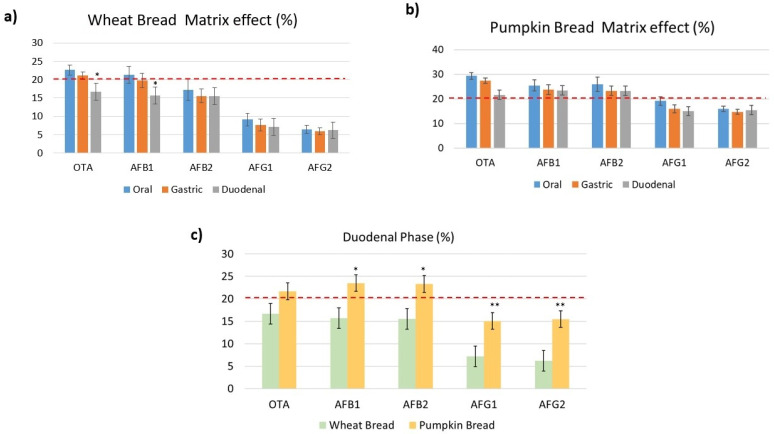
Matrix effect with both studied digested breads in each digested phase. Comparing matrix effect of digested breads in duodenal phase ((*) Represents significant differences (*p* ≤ 0.05) and (**) represents (*p* ≤ 0.01) in (**a**,**b**) from the phases of digestion and in (**c**) from the wheat bread.

The matrix suppression/enhancement effect (ME) was expressed as >0% and <0% indicating matrix suppression and enhancement, respectively. Results within ± 10% were classified as not influenced by the matrix (presented with two black dashed lines in the Figure 3). MEs in digested wheat bread ranged from 22% to 6.4% in the oral phase, 21.1% to 5.9% in the gastric phase and 16.6% to 6.2% in the duodenal phase. However, in digested pumpkin bread the MEs were highest with values ranging from 29 to 15.9% in the oral phase, 27.5 to 14.8% in the gastric phase, and 23.5 to 15.1% in the duodenal phase (Figure 3a,b). AFB_1_ and AFB_2_ presented the highest MEs in digested pumpkin bread, in the duodenal phase (Figure 3c), however, OTA presented higher values of ME than both mycotoxins in the oral and gastric phase. It was observed that during the enzymatic activity, the matrix is less extracted in wheat bread. On average, all the mycotoxins signals were suppressed by the co-eluting matrix. In the literature, none of the consulted articles included and/or evaluate the matrix effect of the different phases in the analytical method. In fact, Massarolo et al. [20] indicate how to evaluate the ME of cornmeal in AFs analysis in this food matrix according to a previous validation, however, neither values are collected for digested phases. 

## 3. Conclusions

According to reported results, to truly estimate levels of mycotoxin exposures in foods, it is necessary to study interactions of mycotoxins with the components of the matrix. Furthermore, a complete validated extraction method is indispensable, and it was observed that few of the studies considered this fact, although none studied this variation along with the digestive system. Therefore, an extraction method was developed and compared for the quantitative determination of five mycotoxins in the oral, gastric and duodenal phases of two types of digested bread (with wheat and pumpkin) for the first time. Under the optimised conditions, the method showed a high extraction efficiency and further validation was carried out. Experiments performed to evaluate the matrix effect, accuracy, and precision demonstrated that the proposed procedure was a sensitive, selective, rapid, robust, and reliable LC–MS/MS method. The digested pumpkin bread presented the highest ME values of 29% of OTA to 15.9% of AFG_2_. The accuracy was above 83% in digested duodenal wheat bread and above 76% in digested duodenal pumpkin wheat bread.

In addition, the developed method was applied to the oral, gastric and duodenal phases of digested bread contaminated at the level of established ML and the results were great at fulfilling the requirements established in Commission Decision (EC) No. 2002/657 [29] demonstrating that it is a reliable procedure to develop next studies of bioaccessibility in bread.

## 4. Materials and Methods

### 4.1. Chemicals and Reagents 

The standard of ochratoxin A (OTA), aflatoxin B_1_ (AFB_1_), aflatoxin B_2_ (AFB_2_), aflatoxin G_1_ (AFG_1_), and aflatoxin G_2_ (AFG_2_) were purchased from Sigma-Aldrich (St. Louis, MO, USA), all of them with a purity of ≥98% (HPLC). Individual stock solutions of mycotoxins were prepared in acetonitrile (AcN) at 500 µg/mL and work solutions in methanol (MeOH) at 100 µg/mL, and maintained at −20 °C in the dark. The final concentration of either MeOH o AcN in the medium was ≤1% (*v*/*v*) as established. 

Methanol (MeOH, HPLC MS/MS grade and OPTIMATM LC/MS grade) and acetonitrile (AcN, HPLC MS/MS grade) were obtained from VWR International (Singapore ) and Fisher Chemical™ (Singapore). Dimethyl sulfoxide was obtained from Fisher Scientific Co., Fisher BioReagents™ (Geel, Belgium). Deionized water (<18, MΩcm resistivity) was obtained in the laboratory using a Milli-QSP^®^ Reagent Water System (Millipore, Beadford, MA, USA). 

To prepare the gastrointestinal solutions KCl 89.6 g/L, KSCN 20 g/L, NaH_2_PO_4_ 88.8 g/L, NaSO_4_ 57 g/L, NaCl 175.3 g/L, NaHCO_3_ 84.7 g/L, urea 20 g/L and Milli-Q water were used. First, a solution of α-amylase (from human saliva Type IX-A, lyophilized powder, 1000–3000 units/mg protein from Sigma-Aldrich) was prepared with 145 mg α-amylase in 100 mL of Milli-Q water. A pepsin solution with 0.5 mg pepsin (1 g in 25 mL HCl (0.1 N) (Pepsin from porcine gastric mucosa, powder, ≥250 units/mg solid, P-7000, Sigma-Aldrich St. Louis, St. Louis, MO, USA) was prepared. Finally, one solution of pancreatin: 1.10 mg (0.1 g Pancreatin (Pancreatin, from Porcine Pancreas, P1750, Sigma-Aldrich St. Louis, MO, USA) and 0.625 g bile salts (Bile extract porcine, B8631, Sigma-Aldrich St. Louis, MO, USA) in 25 mL of NaHCO3 (0.1 N) was prepared. The composition of the saliva was: 10 mL of KCl 89.6 g/L, 10 mL of KSCN 20 g/L, 10 mL of NaH_2_PO_4_ 88.8 g/L, 10 mL of NaSO_4_ 57 g/L, 1.7 mL of NaCl 175.3 g/L, 20 mL of NaHCO_3_ 84.7 g/L, 8 mL of urea (20 g/L) and was completed to 500 mL with Milli-Q water.

### 4.2. Bread Preparation

In this study, two different breads were prepared and they were produced in the laboratory according to the following recipe: 300 g wheat flour, 175 mL water, 20 g fresh yeast (*Saccharomyces cerevisiae*), 10 g sugar and 5 g salt. All ingredients were mixed in a commercial bread maker. The final dough was then cooked at 200 °C for 40 min. Pumpkin bread was prepared as detailed before with the wheat bread with slight modifications to obtain bread with 20% pumpkin. The pumpkin was purchased in a local market and it was a variety of *Cucurbita Maxima Pepo*. It was milled and lyophilized using a lyophilizer Virtis SP SCIENTIFIC sentry 2.0 (Warminster, EE.UU.) and stored at −18 °C until the day of preparation of bread. 

Fortifications were made after the preparation of bread, before the digestion process, adding 1 mL of the working solutions (100 µg/mL) in 10 g of wheat bread.

### 4.3. In Vitro Digestion Procedure

The procedure followed was the in vitro static model based on Brodkorb et al. [32]. This method consists of three digestion phases: oral, gastric and duodenal. Three replicates of each bread were used for the complete digestion assay and put individually sterilized plastic bags (500 mL).

For the oral phase, 10 g of milled bread were mixed with 6 mL of saliva (Section 2.1), 83 mL of milli-Q water, 1 mL of α-amylase solution for 5 min in a shaker. Mastication was simulated 30 s by the Stomacher IUL Instrument (Barcelona, Spain) to homogenize the mixture. The bolus was then put in an opaque Erlenmeyer flask. For the gastric phase, 0.5 mL of pepsin solution was added, and the pH was changed to a pH of 2 with HCl 6 N solution to activate the gastric enzyme. The bolus was incubated for 2 h in heat chamber at 37 °C, under darkness and shaker at 100 rpm (orbital shaker, Infors AG CH-4103, Bottmingen, Switzerland). After the gastric incubation period, 1.10 mL of pancreatin/bile salts solution was added and the pH was adjusted to 6.5 with NaHCO_3_ solution of 1 N. It was incubated for 2 h in a shaker (37 °C, 100 rpm). After the incubation time, pH was adjusted to 7.2 with NaOH at 0.5 N.

After each digestion step, aliquots of each one was taken (5 or 10 mL), put in an ice bath for 10 min to stop enzymatic activity and added enzyme inhibitors, centrifuged at 4500 rpm, 4 °C, 5 min and stored until the determination of the mycotoxins and the duodenal bioaccessibility. The duodenal phase that remained in the Erlenmeyer conical flask was centrifuged at 4000 rpm, 4 °C, 10 min and then filtered. All supernatant obtained was frozen at −20 °C. Additionally, enzyme inhibitors were used, sufficient amounts of enzyme inhibitors against target digestive enzymes are strongly recommended. A Bowman–Birk inhibitor (BBI) (Sigma-Aldrich, cat. no. T9777) was used as inhibitor of both trypsin and chymotrypsin; for amylase, inhibition snap freezing treatment and inactivation by extraction solvent; and Pepstatin A (Sigma-Aldrich, cat. no. P5318) was used for pepsin inhibition. A total of 100 μL of a BBI solution (0.05 g/L) and pepstatin A at 0.5–1.0 μM final concentration was used.

### 4.4. Sample Analysis

#### 4.4.1. Extraction of AFs and OTA from the Simulated Physiological Fluids

The extraction of the mycotoxins in simulated physiological fluid was carried out in each three steps of the digestive process. Three aliquots of the digested phases were fortified at three levels (0.25, 0.5 and 1 µg/mL) and in triplicate. The extraction was performed with a liquid–liquid extraction according to El Jai et al. [33] with slight modifications using ethyl acetate. The fortified aliquot was centrifuged and the upper layer was transferred to a falcon tube. After that, 5 mL of ethyl acetate was added twice, shaken and centrifuged using Eppendorf centrifuge 5810R (Eppendorf, Hamburg, Germany). The upper layer was placed in 15 mL PTFE centrifuge tubes and was evaporated to dryness at 35 °C with a soft stream of nitrogen using a multisample TurboVap LV Evaporator (Zymark, Hoptkinton, MA, USA). 

#### 4.4.2. LC–MS/MS Analysis

Before LC–MS/MS analysis, the dry residues were reconstituted to a final volume of 0.5 mL with methanol/water (70:30, *v*/*v*) and filtered through a 13 mm/0.22 μm nylon filter purchased from Análisis Vínicos S.L (Tomelloso, Spain).

The analysis was performed using an LC-MS/MS system consisting of an LC Agilent 1200 using a binary pump and an automatic injector, and coupled to a 3200 QTRAP^®^ABSCIEX (Applied Biosystems, Foster City, CA, USA) equipped with a Turbo-VTM source (ESI) interface. The chromatographic separation of the analytes was conducted at 25 °C with a reverse-phase analytical column Gemini^®^NX-C18 (3 μM, 150 × 2 mm ID) and a guard column C18 (4 × 2 mm, ID; 3 μM). Mobile phase was a time programmed gradient using methanol as phase A (0.1% formic acid and 5 mM ammonium formate), and water as phase B (0.1% formic acid and 5 mM ammonium formate). The following gradient was used: equilibration for 2 min at 90% B, decrease linearly to 20% of phase B in 3 min, maintain 20% of phase B for 1 min, decrease linearly from 20 to 10% of phase B in 2 min, maintain 10% of phase B for 6 min, decrease to 0% B in 3 min, maintain 100% A for 1 min, finally increase linearly from 0 to 50% B in 3 min, return to initial conditions (90% B) in 2 min and maintain during 2 min. The flow rate was 0.25 mL/min in all steps. Total run time was 21 min. The injection volume was 20 µL.

With regard to mycotoxin analysis, the QTRAP system was used as triple quadrupole mass spectrometry detector (MS/MS). The Turbo-V™ source was used in positive mode to analyze AFs and OTA with the following settings for Source/Gas Parameters: Vacuum Gauge (10e-5 Torr) 3.1, curtain gas (CUR) 20, ionspray voltage (IS) 5500, source temperature (TEM) 450 °C, ion source gas 1 (GS1), and ion source gas 2 (GS2) 50. The precursor ions (Q1), product ions (Q3), and collision energies (CE) are shown in Table 3. The entrance potential (EP) was the same for all analytes, 10 V. Acquisition and processing data were performed using Analyst^®^software, version 1.5.2 (AB SCIEX LP, Concord, ON, Canada). The fragments monitored (retention time, quantification, ion and confirmation ion) and spectrometric parameters (declustering potential, collision energy, and cell exit potential) used are that performed previously by Juan, et al. [34].

### 4.5. Method Validation Procedure for Mycotoxins Analysis in Each Digested Phase

LC–MS/MS method was validated following the validation procedure by El Jai et al. [33], based on the guidelines and recommendations defined by the Commission Decision (EC) No. 2002/657 [26] and Regulation (EC) No. 401/2006 [35]. Linearity, limit of quantification (LOQ), limit of detection (LOD), trueness, and precision (repeatability and reproducibility) were assessed. Since digested bread samples (control bread and spiked bread) were performed in triplicate, mycotoxins were analyzed in the three digested phases. A mix of the triplicate digestion in each three phases digested was used to obtain the calibration curve. Each digested sample was spiked with a multi-standard working solution at analyte-dependent concentration levels 2.5, 5 and 10 μg/kg, level L1, level L2 and level L3, respectively. Matrix-matched standards, e.g., blank samples which were digested and extracted after sample preparation, were prepared for the three concentration levels mentioned. The experiments were performed over two more days (*n* = 6) for the reproducibility study. 

Calibration curves were generated on six concentration levels. To assess method linearity, calibration curve equations and coefficients of determination (R2) using a linear regression model were determined. The lowest calibration point represented the LOQ which was estimated in preliminary studies and calculated based on the S/N ratios. According to the International Union of Pure and Applied Chemistry (IUPAC) guidelines, S/N values of at least 10 were required for the quantifier as well as qualifier transitions. Repeatability (RSDr) and within-laboratory precision (RSDR) were determined on the three lowest calibration points to estimate the precision of the method. Sets of fortified samples were prepared at each concentration level, analyzed, and concentrations were calculated. These steps were repeated on two other occasions and the RSDr was represented in terms of relative standard deviations (RSDs). For determination of the RSDR, sets of six replicates were prepared on two other days by different operators, analyzed in different runs, and RSDs were calculated. Absolute recovery rates (RE, *n* = 6) were evaluated using the ratios of matrix-spiked to matrix-matched standards. The matrix effect (ME) evaluation was performed by the calculation of the ratios of matrix-matched to solvent-only standards. The mean of the MEs determined at the three lowest spiking levels was visualized in negative values for matrix suppression and positive values for matrix enhancement.

### 4.6. Statistical Analysis

Statistical analysis of data (correlation analysis, multiple linear regression analysis, Student’s *t*-test) was carried out using Microsoft Excel 2015 statistical software package. Data were expressed as mean ± SD of three independent experiments. The statistical analysis of the results was performed by Student’s t-test for paired samples. Differences with respect to the control group were statistically analyzed using ANOVA followed by the Tukey HSD post hoc test for multiple comparisons; *p* ≤ 0.05 was considered statistically significant.

## Figures and Tables

**Table 2:** Validation data of the extraction method in duodenal digested wheat bread.

Mycotoxin	Repeatability (R ± SD, %)			Reproducibility (R ± SD, %)		
	L1	L2	L3	L1	L2	L3
AFB_1_	102 ± 1	110 ± 4	103 ± 9	106 ± 4	101 ± 4	107 ± 9
AFB_2_	92 ± 5	105 ± 6	108 ± 12	96 ± 3	90 ± 2	94 ± 10
AFG_1_	86 ± 3	91 ± 4	85 ± 8	106 ± 8	103 ± 5.3	105 ± 11
AFG_2_	97 ± 9	87 ± 6	83 ± 8	107 ± 12	94 ± 7.9	108 ± 10
OTA	92 ± 13	103 ± 17	104 ± 18	93 ± 12	106 ± 13	98 ± 12

R: Mean recovery; SD: Standard deviation; level L1: 2.5 μg/kg, level L2: 5 μg/kg and level L3: 10 μg/kg.

**Table 3:** Mass spectrometry parameters data for identification of studied mycotoxins.

Mycotoxin	Rt * (min)	Quantitation Transition			Qualifier Transition		
		Q_1_ (*m*/*z*)	Q_3_ (*m*/*z*)	CE (eV)	Q_1_ (*m*/*z*)	Q_3_ (*m*/*z*)	CE (eV)
AFB_1_	7.81	313	241	41	313	284	39
AFB_2_	7.69	315	259	39	315	286	33
AFG_1_	7.57	329	311	29	329	243	39
AFG_2_	7.47	331	245	39	331	313	27
OTA	8.75	404	102	97	404	239	27

* Rt: retention time; Q_1_: precursor ions; Q_3_: product ions; CE: collision energy.

## Data Availability

Not applicable.
